# A True Metasurface Antenna

**DOI:** 10.1038/srep19268

**Published:** 2016-01-13

**Authors:** Mohamed El Badawe, Thamer S. Almoneef, Omar M. Ramahi

**Affiliations:** 1 Department of Electrical and Computer Engineering, Waterloo, N2L3G1, Canada

## Abstract

We present a true metasurface antenna based on electrically-small resonators. The resonators are placed on a flat surface and connected to one feed point using corporate feed. Unlike conventional array antennas where the distance between adjacent antennas is half wavelength to reduce mutual coupling between adjacent antennas, here the distance between the radiating elements is electrically very small to affect good impedance matching of each resonator to its feed. A metasurface antenna measuring 1.2*λ* × 1.2*λ* and designed to operate at 3 GHz achieved a gain of 12 dBi. A prototype was fabricated and tested showing good agreement between numerical simulations and experimental results. Through numerical simulation, we show that the metasurface antenna has the ability to provide beam steering by phasing all the resonators appropriately.

The hypothesis of a medium that simultaneously have negative permittivity and permeability in a given frequency and the experimental realization of this hypothesis brought great interest to the scientific and engineering communities^1^,^2^. Artificial materials have originated numerous applications for electromagnetic research, including advanced lenses^3^,^4^, cloaking^5^, energy harvesting^6^,^7^,^8^, and material based antennas^9^. Metamaterials are often manufactured as a periodic ensemble of conducting elements such as metallic rings and roads or even spherical particles, which collectively act as an effective electromagnetic medium^2^,^10^ with effective permittivity and permeability. Metamaterials have different classes: negative index, single index, bandgap^11^, and metasurface, the latter of which has wide potential applications, such as absorbers^12^,^13^, harvesters^14^, and detector of microwave radiation^15^.

Antennas with dimensions smaller than the wavelength of free space are typically referred to as electrically-small antennas. This type of antennas has much interest especially in wireless communication systems requiring small form factor where it can be applicable in personal communication systems (e.g. headphones, watches, and pagers), military applications, unmanned aerial vehicles, and more^9^,^16^. To have light weight, low profile and inexpensive antennas which would be efficient, easy to build, and easy to integrate with other communication devices, as well as having a desirable bandwidth and gain, important and complicated steps have to be considered in the design procedure. One of these steps is impedance matching of the antenna to the feed circuitry^9^,^16^,^17^. Antenna arrays are topological arrangements of antennas that are distanced and phased in such a way to produce higher gain and/or beam scanning in various directions. The array, however, brings a higher-order complexity to the design procedure especially if it needs to be fed by one point^18^,^19^. Moreover, electrically-small antennas have fundamental theoretical limitations on gain, radiation efficiency, and bandwidth where the antenna size is inversely correlated to the quality factor and the radiation loss^20^,^21^.

Over the past few years, metamaterials have been used in many different ways to miniaturize antennas, such as complementary split-ring resonator (CSRR) loaded antenna^22^,^23^, slotted complementary split-ring resonator (SCSRR)^24^, and artificial magnetic materials with fractal Hilbert inclusions^25^. Moreover, metamaterials have been used as an effective medium to enhance the conventional antennas gain^26^ and directivity^27^ rather than consider it as the main radiators. Metamaterial based antennas were proposed earlier to develop small antennas by miniaturizing the antenna size while maintaining good antenna performance^9^,^17^,^28^.

In this work, we present first generation of metasurface antennas operating in the microwave regime. Unlike classical and traditional antennas where radiation is initiated by enhanced concentration of current density by exploiting wavelength-based resonance, the metasurface antenna concept presented in this work is based on an ensemble of electrically-small resonators whose resonance strongly resembles classical circuit resonance, however with the difference that the impedance elements have spacial dimension thus allowing coupling to external fields. The ensemble of the electrically-small resonators gives high degree of freedom for controlling the magnitude and phase of the current over a large portion of the metasurface. While each of the resonators does not constitute a good radiator if it were considered individually, the ensemble of elements acting together provide excellent radiation characteristics facilitated by good impedance matching due to tailored inter-element coupling^29^.

## Design Methodology

The particular electrically-small resonators chosen for demonstrating the metasurface antenna concept are the electrical ring resonator (ERR) reported earlier in the literature^30^. Arranging a periodic array of these symmetric metallic elements will create a class of subwavelength particles which exhibit a strong resonance response to the electrical field and negligible response to the magnetic field. We propose a feed network to connect all radiators to one feed point. The feed network was designed in a way to match the radiators impedances to the feed impedance to ensure optimal antenna gain and bandwidth.

**Figure 1:**
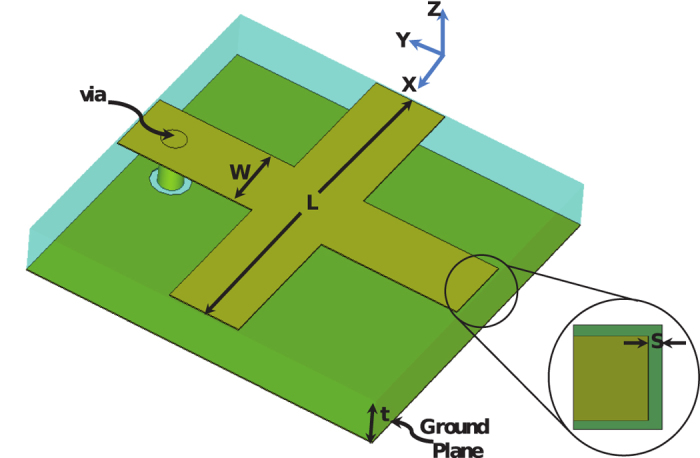
A schematic showing the proposed unit cell of a metasurface antenna and its optimized dimensions as well as the placement of the via.

The unit cell considered in this work is the cross resonator element reported earlier to develop high absorbing surface is shown in Fig. 1^30^. Cross strips are etched on one side of a 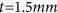 thickness Rogers TMM10i substrate with a dielectric constant of 9.9 and a loss tangent of 0.002 while the other side of the substrate is kept metalized serving as a ground plane. The geometric dimensions of the cell are optimized to achieve minimum reflection coefficient 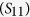 occuring at 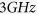, 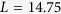 mm, 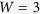 mm, 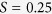 mm, and copper thickness is 35 *μ*m.

**Figure 2:**
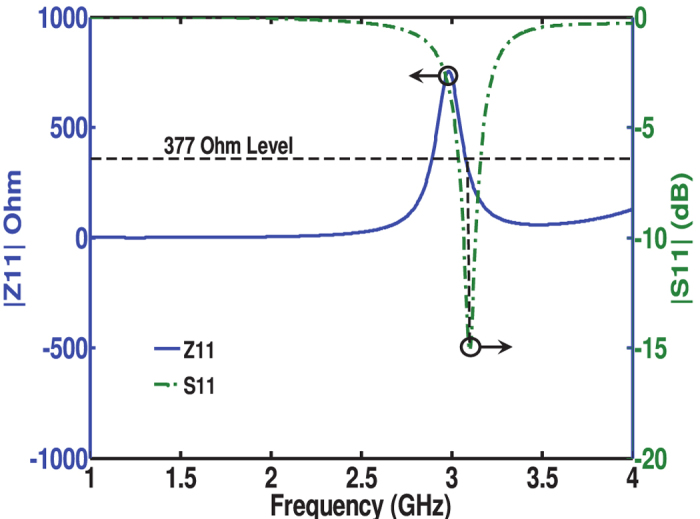
Surface impedance of the metasurface at the resonance frequency. The blue solid line gives the magnitude of the surface impedance for a normally incident plane wave and the green dashed-dotted line gives the magnitude of the reflection coefficient. The simulation was performed by placing the ERR at the center of a waveguide with perfect electric wall in the x-z plane and perfect magnetic wall in the x-y plane to realize TEM mode excitation in the z-direction. The antenna was placed parallel to the x-y plane while the two open ports were in the z-direction. The boundary conditions were chosen to force the incident electric and magnetic fields to be parallel to the structure surface.

The Commercial program CST Microwave Studio was used to model the proposed antenna^31^. In order to examine the ERR behavior, to simulate the S-parameters response to achieve minimum reflection coefficient 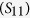, and to find the surface impedance of the cell at 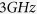, the ERR was placed in the center of a waveguide with perfect electric wall in the 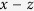 plane and perfect magnetic wall in the 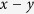 plane to realize TEM mode excitation in the z-direction (the antenna was placed parallel to the x-y plane while the two open ports were in the z-direction). The boundary conditions were chosen to force the incident electric and magnetic fields to be parallel to the structure surface. From the impedance results obtained, shown in Fig. 2, it is evident that the media provided a surface having an impedance of 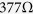 at the operating frequency. This indeed gives validation that the antenna surface acts as a metasurface since it can be represented by an equivalent surface with a homogenous permittivity and permeability.

**Figure 3:**
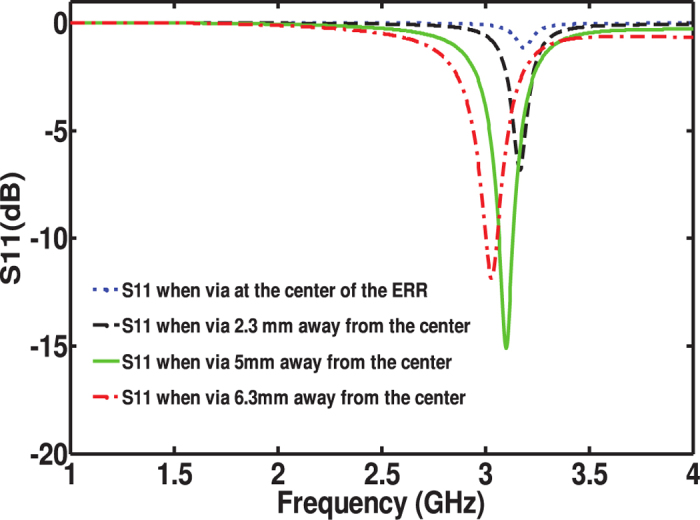
Dependence of |(*S*_11_)| on the via position.

A via with diameter of 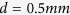 was chosen due to fabrication constraints which required a via size of 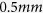 to channel the current to the load. The optimal via position is heuristically optimized and it is shown in Fig. 3. From the results, a via position of around 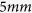 away from the center of the cross resonator and a via size of 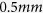 provided a reflection coefficient of less than 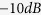 at the operating frequency. The load resistance was chosen to be 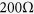 to simplify the feeding network design. By doing so, one can design the feeding network with minimum number of stages to arrive at the impedance of the feed port of 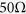. Of course, other feed networks could be chosen, but it is advantageous to choose impedance levels that avoid the need for very thin transmission line widths which are challenging to fabricate and thus can lead to measurements uncertainties.

**Figure 4:**
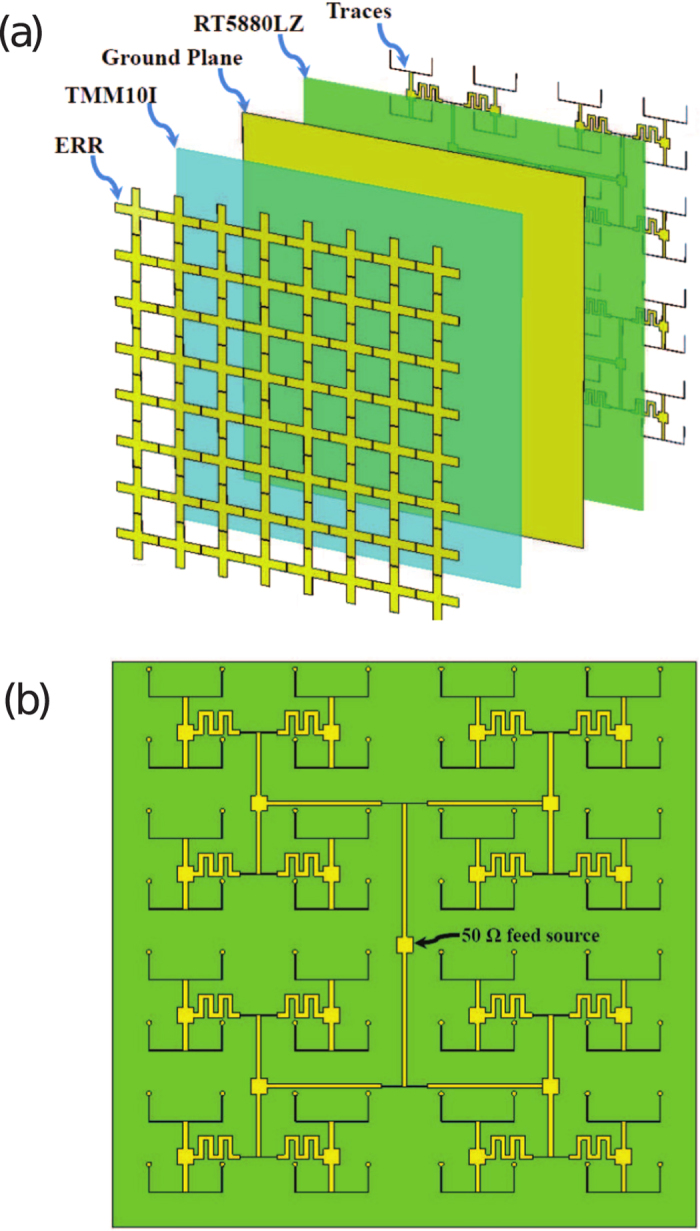
Architecture of the proposed metasurface antenna. (**a**) Diagram of the antenna elements is shown as exploded view, including the electrical ring resonators, Rogers TMM10I substrate as first substrate, ground plane (copper), Rogers RT5880LZ as second substrate, and the transmission line traces. (**b**) Symmetrical configuration of the corporate fed array (64-element).

The metasurface antenna presented here is an array of 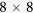 ERR elements periodically arranged on a square substrate with total dimensions 12 cm × 12 cm as shown in Fig. 4(a). Each resonator has a 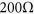 input impedance which was adjusted by careful tuning of the inter-element spacing and geometrical dimensions of the resonator. All radiators were connected to one feed port having a 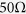 input impedance. A matching circuit based on microstrip transmission lines is used to connect all the elements to one 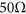 feed port. Unlike conventional antennas where the antennas are typically separated by a distance of half wavelength which allow for sufficient space within the antenna structure to build the feeding network for the metasurface antenna, the distance between the radiating elements is very small which cannot fit the feeding network. Therefore, a 1 mm thick Rogers RT5880LZ substrate with a dielectric constant of 1.96 and a loss tangent of 0.002 (at the chosen operating frequency) was added to the structure. Figure 4(b) shows the corporate feed network that was used to connect all radiators to a 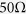 feed port. The width and length of the traces were calculated using microstrip transmission line equations^32^. In this arrangement, all radiating elements were fed in phase in order to achieve highest gain in the z-direction (broadside).

## Simulation Results

**Figure 5:**
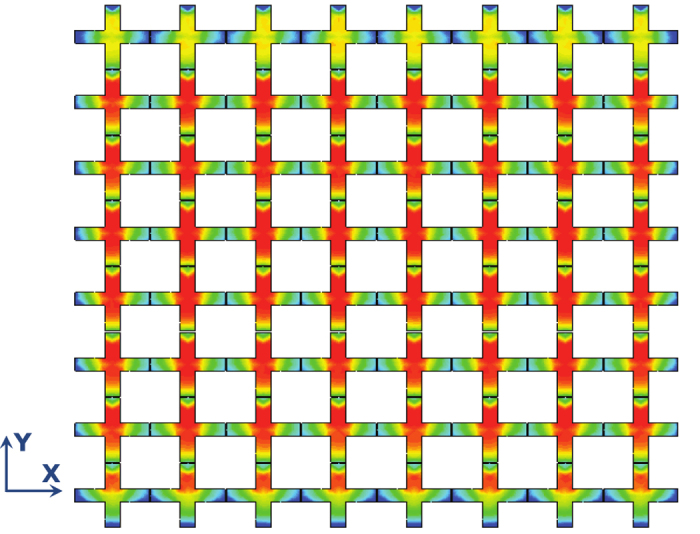
Current distribution on the ERRs at the resonance frequency of 2.97 GHz. The highest intensity (red) corresponds to 138 A/m and the lowest intensity (blue) corresponds to 0 A/m.

**Figure 6:**
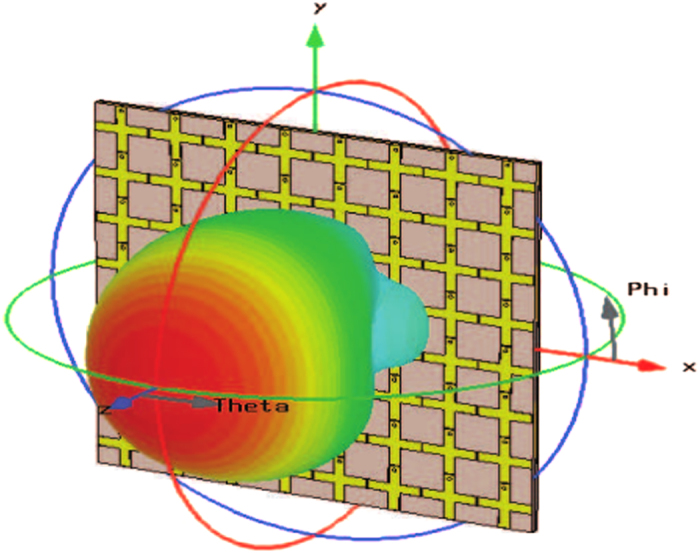
The simulated 3D gain radiation pattern of the metasurface antenna. The highest intensity (red) corresponds to 11.7 dBi and the lowest intensity (blue) corresponds to −28.3 dBi

Figure 5 shows the simulated current which is observed to be distributed uniformly over the metasurface. As shown, the current is high in one direction of the ERR and low in the other direction because each element was excited by a via placed at one side of the y-directed arm of the resonator (see Fig. 1). The asymmetry in current distribution on the ERRs at the top and bottom edges of the metasurface is because the input impedance of those ERRs are slightly different from 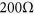. The simulated 3D gain radiation pattern of the metasurface antenna is shown in Fig. 6. The gain, directivity, and the radiation efficiency at 2.97 GHz were found to be 11.7 dBi, 12 dBi, and 91.5%, respectively. The uniformity of the current distribution and its highly directional intensity is responsible for the achieved high antenna directivity.

## Experimental Verification

**Figure 7:**
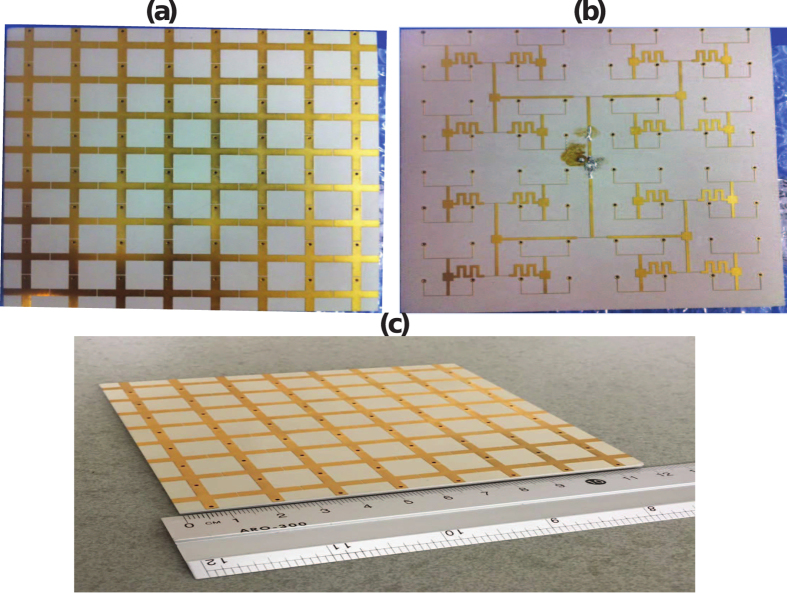
Photograph of the fabricated metasurface (a) top view (b) bottom view and (c) perspective view.

**Figure 8:**
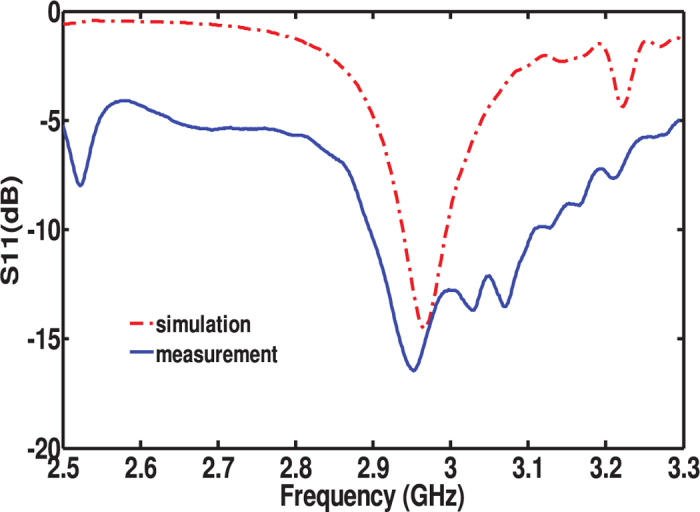
Simulated and measured results of the return loss of the metasurface antenna.

An 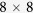 elements metasurface antenna was fabricated based on the simulated design. All 64 radiators were connected together to a 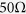 feed point as shown in Fig. 7(b). A vector network analyzer was used to measure the return loss 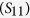 at the structure feed point. The measurement results were compared to the simulation results which are presented in Fig. 8. Good agreement is observed between simulation and measurement with approximately 15 MHz shift in the resonance frequency. We observe that the measurement gives wider bandwidth than the simulation.

**Figure 9:**
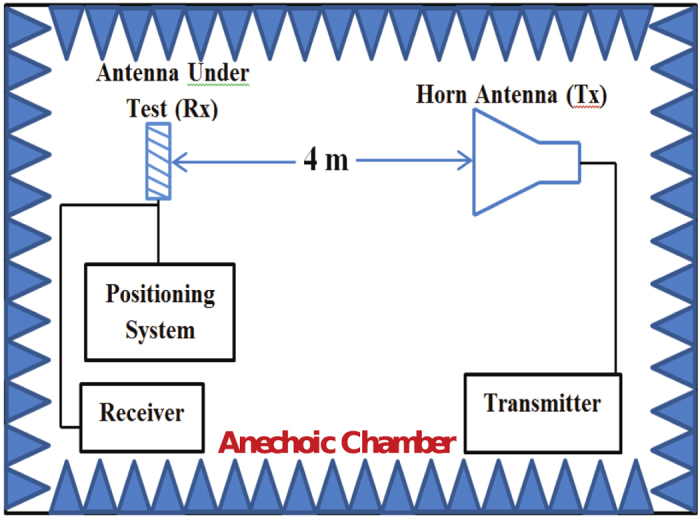
The gain measurement setup used in the experiment.

**Figure 10:**
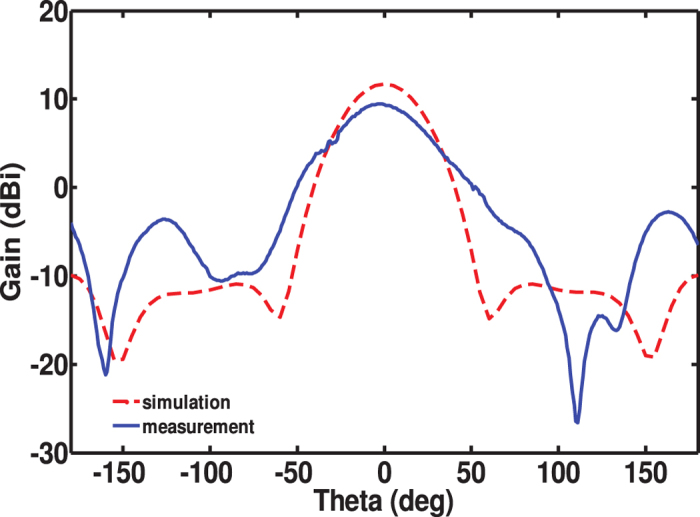
Simulated and measured radiation pattern.

Measurements of gain were performed in an anechoic chamber, see Fig. 9. The metasurface antenna was placed approximately four meters away from a standards horn antenna to ensure the incident field at the metasurface antenna is a plane wave. Considering the linear polarization of the antenna under the present feed design, the metasurface antenna was positioned such that the radiated electric field was parallel to the section (strip) of the ERR resonators in which the vias were located as shown in the Fig. 1. The gain was measured over a frequency range from 2 GHz to 4 GHz in 50 MHz increments with highest gain of 9.4 dBi obtained at 2.95 GHz. Figure 10 shows the measurement and simulation gain plots.

## Discussion

Several factors contributed to the slight variation between the experimental and simulation results. The second layer of the structure was chosen to have very low dielectric constant (RT5880LZ) because the transmission line width needed to achieve specific impedance is highly sensitive to the dielectric constant of the material. The transmission line widths were calculated using standard microstip transmission line equations which were based on approximations. Furthermore, the RT5880LZ substrate was difficult to etch resulting in small miss-alignments and line-width discrepancies in the final design. Finally, we note that the positioning of the antennas was done manually, hence, misalignment between the transmitting and receiving antenna could have affected the accuracy of the antenna gain measurements.

**Figure 11:**
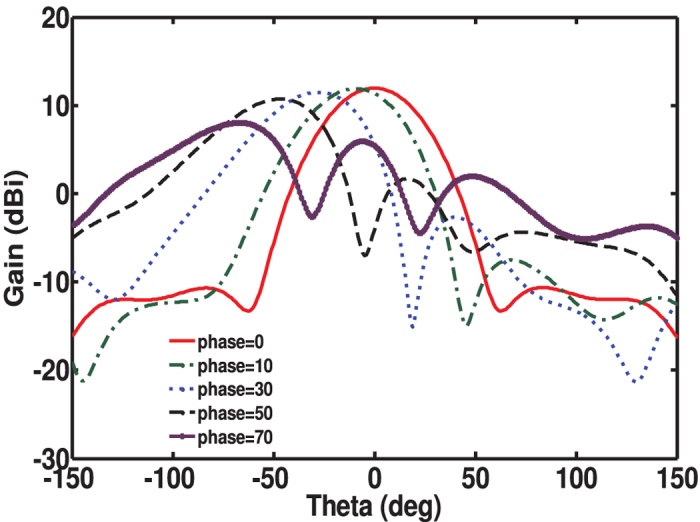
Scanning potential of the metasurface antenna obtained using numerical simulation. Correspondence between the progressive inter-element phase and the scan angle with maximum gain is as follow: Phase = 10° corresponds to *θ* = 9°, Phase = 30° corresponds to *θ* = 28°, Phase = 50° corresponds to *θ* = 47°, and Phase = 70° corresponds to *θ* = 66°.

The gain of the metasurface antenna can be increased incrementally by adding additional elements. For instance, through numerical simulation, a 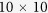 elements metasurface antenna achieved a gain of 13.5 dBi. Additionally, the antenna can be used for scanning by varying the phase across the electrically-small resonators comprising the metasurface. Since the resonating elements were positioned very tightly next to each other, strong coupling between them precludes the use of antenna array theory to predict the scan angle. To scan in *θ* (see Fig. 6), the elements along the x-axis were phased progressively while the elements along the y-axis had constant phase. Figure 11 shows the scanning potential of the 8 × 8 element metasurface used in this work. While the gain is observed to slightly decrease with increasing scan angle, the scanning is achieved despite the relatively small footprint of the antenna. To create progressive phase shift across elements, as for instance to achieve steering the beam in a different direction as discussed, one needs to add a microstrip line segment to each element where the phase is needed. The length of the transmission line segment is chosen to introduce the required phase. The metasurface antenna is also capable of scanning in *θ* and 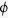 by simultaneously phasing the elements along the x- and y-directions. Finally, we note that the metasurface antenna can be used on irregular (non-Cartesian) platforms such as curved surfaces of air vehicles or human body. Fundamentally, the radiation pattern can be tailored to achieve optimum performance by optimization of the phase of the N × N elements. Such high degree of design freedom is not present in classical single element antennas. In fact, the tailoring of the phase to achieve desired gain in specific direction has resemblance to the concept of metamaterial huygens’ surfaces^33^ and designer metasurface for flat optics^34^. In those works, the metasurfaces provided control of electromagnetic wavefronts across electrically-thin layers. Here, the metasurface antenna generates the desired wavefront by direct manipulation of the phase of each element comprising the metasurface.

## Conclusion

In conclusion, we introduced a new concept for antennas design based on a metasurface. While each electrically-small resonator/element of the metasurface is a poor radiator if acting alone, the ensemble of elements work in synergy, including balance of the impedance of each element, to create highly directional antenna. We demonstrated the metasurface concept using simulation and laboratory measurements. The metasurface antenna has strong potential in variety of traditional and non-traditional applications where its flexible design (high degree of optimization freedom) facilitates its use on variety of platforms.

## Additional Information

**How to cite this article**: Badawe, M. E. *et al.* A True Metasurface Antenna. *Sci. Rep.* **6**, 19268; doi: 10.1038/srep19268 (2016).
